# Using NMR spectroscopy to investigate the role played by copper in prion diseases

**DOI:** 10.1007/s10072-020-04321-9

**Published:** 2020-04-24

**Authors:** Rawiah A. Alsiary, Mawadda Alghrably, Abdelhamid Saoudi, Suliman Al-Ghamdi, Lukasz Jaremko, Mariusz Jaremko, Abdul-Hamid Emwas

**Affiliations:** 1 King Abdullah International Medical Research Center (KAIMRC), Jeddah, Saudi Arabia/King Saud bin Abdulaziz University for Health Sciences (KSAU-HS), Jeddah, Saudi Arabia; 2 Biological and Environmental Science and Engineering (BESE), King Abdullah University of Science and Technology (KAUST), Thuwal, Kingdom of Saudi Arabia; 3 Oncology, Ministry of National Guard Health Affairs, Jeddah, Saudi Arabia. King Abdullah International Medical Research Center (KAIMRC), Jeddah, Saudi Arabia/King Saud bin Abdulaziz University for Health Sciences (KSAU-HS), Jeddah, Saudi Arabia; 4 Imaging and Characterization Core Lab, King Abdullah University of Science and Technology (KAUST), Thuwal, Kingdom of Saudi Arabia

**Keywords:** Prion disease, Prion protein, Copper-binding site, NMR, Paramagnetic ions, Neurodegenerative disorder, Protein stability

## Abstract

Prion diseases are a group of rare neurodegenerative disorders that develop as a result of the conformational conversion of normal prion protein (PrP^C^) to the disease-associated isoform (PrP^Sc^). The mechanism that actually causes disease remains unclear. However, the mechanism underlying the conformational transformation of prion protein is partially understood—in particular, there is strong evidence that copper ions play a significant functional role in prion proteins and in their conformational conversion. Various models of the interaction of copper ions with prion proteins have been proposed *for the* Cu (II)-*binding*, cell-surface *glycoprotein known as prion protein* (PrP)*.* Changes in the concentration of copper ions in the brain have been associated with prion diseases and there is strong evidence that copper plays a significant functional role in the conformational conversion of PrP. Nevertheless, because copper ions have been shown to have both a positive and negative effect on prion disease onset, the role played by Cu (II) ions in these diseases remains a topic of debate. Because of the unique properties of paramagnetic Cu (II) ions in the magnetic field, their interactions with PrP can be tracked even at single atom resolution using nuclear magnetic resonance (NMR) spectroscopy. Various NMR approaches have been utilized to study the kinetic, thermodynamic, and structural properties of Cu (II)-PrP interactions. Here, we highlight the different models of copper interactions with PrP with particular focus on studies that use NMR spectroscopy to investigate the role played by copper ions in prion diseases.

**Electronic supplementary material:**

The online version of this article (10.1007/s10072-020-04321-9) contains supplementary material, which is available to authorized users.

## Background

**Fig. 1:**
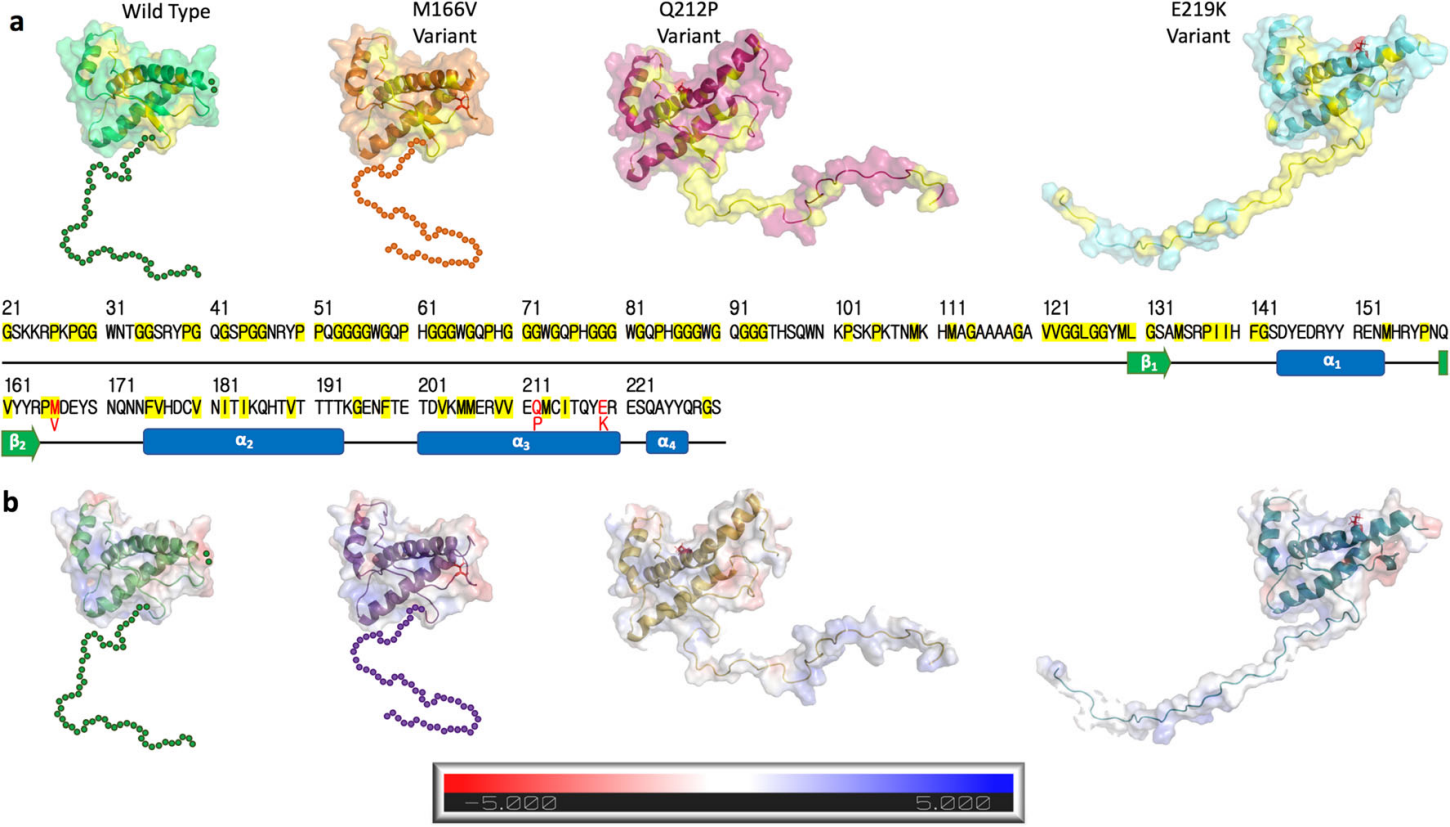
**a** Hydrophobic regions of PrP highlighted in yellow. **b** Comparison of the electrostatic surface between the wild-type PrP and variants. The human PrP protein consists of 253 amino acids. PrPC contains an octapeptide repeat-containing unfolded domain at the N-terminal tail, three α-helices (α1, α2, and α3), antiparallel β-sheets (β1 and β2), and a GPI-anchor signal at the C-terminal tail

Prion diseases are a family of rare and progressive neurodegenerative disorders that develop as a result of the conformational conversion of the normal form of the transmissible prion protein (PrP^C^) into the disease-associated form (PrP^Sc^) [1]. These diseases usually take many years to develop; during the incubation period, the disease advances asymptomatically in the brain until initiation of nervous system degeneration and subsequent death [2]. Human (Hu) PrP^C^ is a 209-residue glycoprotein that is attached by a C-terminal glycosylphosphatidylinositol (GPI) to the outer leaflet of the plasma membrane of a brain cell. Prion proteins are highly conserved among mammals [3, 4], where the general structure of globular domain protein, PrP^C^ contains three α-helices and a two-strand antiparallel β-sheets, an NH_2_-terminal tail consisting of an octapeptide repeat-containing unfolded domain, and GPI attached to the short COOH-terminal tail [5]. Figure 1 shows the structures of various prion proteins.

**Fig. 2:**
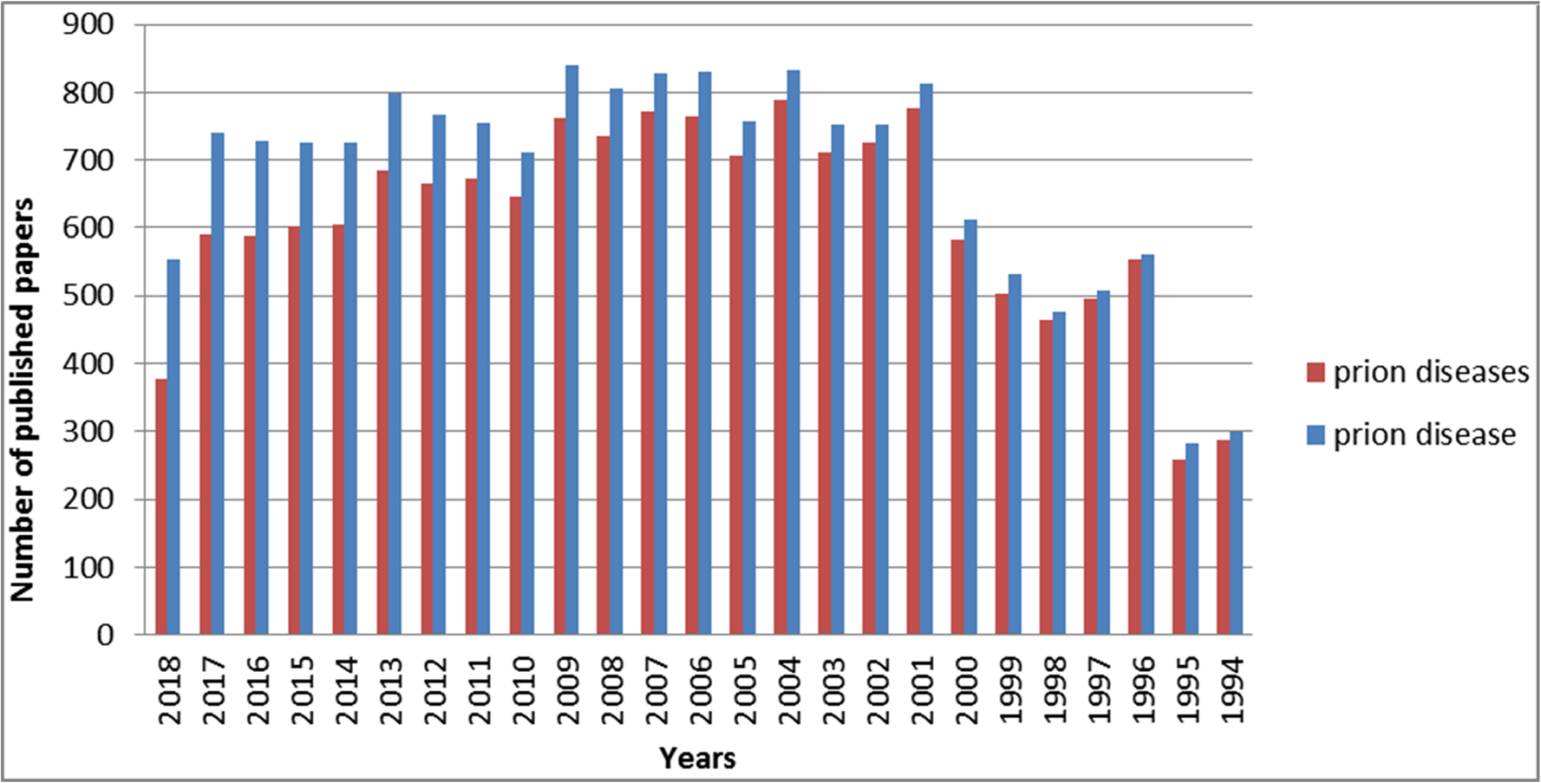
Number of published papers using PubMed search engine. The PubMed database was searched for prion diseases (Red) and prion disease (Blue) keywords over the last 25 years. Last updated on September 25, 2018

Repeated published reports noted that copper may play a significant role in the conversion of PrP^C^ to PrP^Sc^ [6–9] (Fig. 2). Moreover, several reports have shown that cellular prion protein (PrP) may play a crucial role in the redox control of the neuronal environment and in the regulation of copper metabolism in a manner that contributes to disease pathology [7, 10–12]. The concentration of copper in humans varies in different organs. A high copper concentration is found in the liver, brain, kidney, and heart [13]. In these organs, copper is essential for the function of several enzymes, including cytochrome C oxidase, catalase, dopamine hydroxylase, uricase, tryptophan dioxygenase, lecithinse, and other monoamine and diamine oxidases as well as superoxide dismutase (SOD) [14–18]. These enzymes are important in oxidation-reduction reactions, transport of oxygen and electrons, and protection of the cell from oxygen radicals [19, 20]. Changes in copper ion concentrations in the brain are associated with several neurological diseases including prion diseases [21–24]. Gasperini et al. showed that PrP^C^ and copper jointly inhibit N-methyl-d-aspartate receptors (NMDAR) and prevent cell death, thus suggesting a positive role for copper in disease treatment [12]. They also showed that PrP^C^ and copper cooperatively protect neurons from insults and exert neuroprotective effects [12].

Advances in medical research and technology, such as nuclear magnetic resonance (NMR) spectroscopy and imaging, have contributed enormously to the detection and management of prion diseases [25–36] as was successfully demonstrated in the detection and description of Creutzfeldt-Jakob disease (CJD) [30]. In particular, NMR spectroscopy provided the first three-dimensional (3D) structure of the folded mouse prion protein domain PrP [12, 37–146]. Recently, structures of other PrP-associated diseases were resolved using NMR spectroscopy [147]. In addition, NMR enabled the investigation of the dynamic equilibria between monomeric and oligomeric misfolded states of mammalian PrP [148].

## Prion disease

Prion diseases, such as human prion diseases, are a group of progressive neurodegenerative disorders caused by conformational conversion of the α-helix-rich isoform of the prion protein (PrP^C^), which is the normal form, into the β-sheet rich isoform (PrP^Sc^), which is the disease-associated form [1, 149, 150]. Abnormal folding of the protein (PrP^Sc^) leads to brain damage and causes high fatality rates in both humans and animals [151–166]. However, the pathogenic mechanism that triggers this abnormal folding leading to prion diseases remains unknown. Prion diseases may take many years to develop with long incubation periods [2, 149]; during this time, the disease grows asymptomatically in the brain until the initiation of nervous-system degeneration and resulting death [2]. The infection causes brain atrophy, spongiform encephalopathy, and cerebellar degeneration. Although prion diseases are rare, they remain an important public health issue requiring attention to their management [167].

**Fig. 3:**
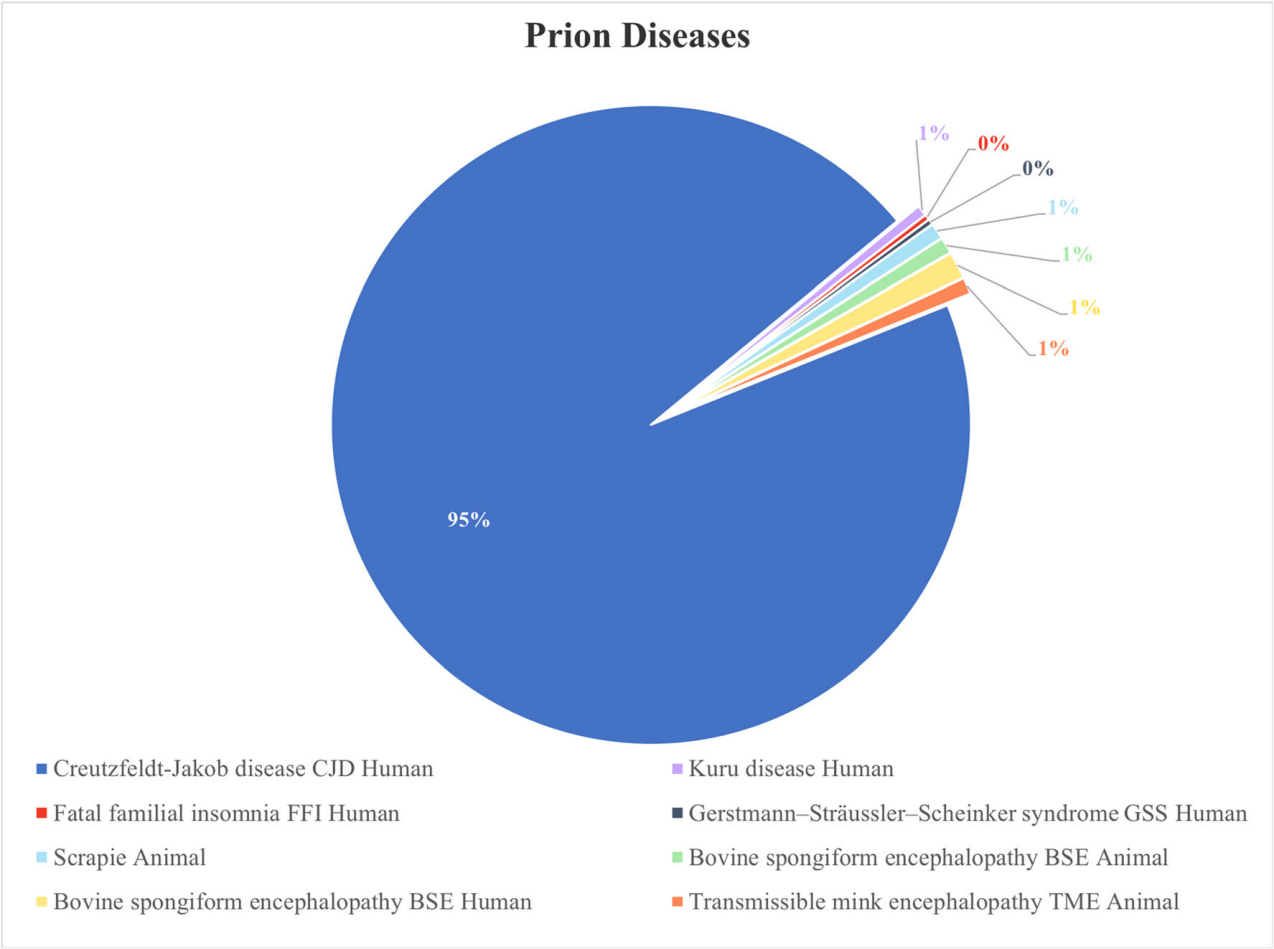
The occurrence ratio of the most common prion diseases

Prion diseases can be contracted through sporadic, genetic, and infectious routes [168–171]. An individual who contracts a prion disease sporadically is exposed to unknown risk factors that vary from one region to another [169]. Some people and animals can inherit prion diseases from their parents, whereas others acquire it from contaminated animal products and feed. The most common types of animal prion diseases are scrapie, bovine spongiform encephalopathy (mad cow disease), and transmissible mink encephalopathy [172, 173]. Examples of human prion diseases are Creutzfeldt-Jakob disease (CJD), Kuru, fatal familial insomnia (FFI), and Gerstmann-Sträussler-Scheinker syndrome (GSS) [147, 174, 175]. Neurological clinical presentations and diagnosis vary among the different human prion diseases. Research has shown that Kuru disease has been eradicated, where it acquired through consumption of the brains of infected humans killed by the disease during the practice of funerary cannibalism [176]. FFI is an autosomal illness characterized by lesions in the thalamus of the brain. GSS is associated with the pathological Q212P mutation, and, like CJD, results in progressive dementia [147, 177]. CJD is associated with mutation in the gene encoding the prion protein [178] and the most common and fatal prion disease (Fig. 3), with about 90% of affected individuals dying within a year of diagnosis. Early symptoms include poor coordination, visual disturbance, and memory problems; later symptoms include blindness, weakness, involuntary movement, and finally coma**.** Additional file 1: Table S1 summarizes the similarities and differences among the various human prion diseases.

## Copper ions in nervous system development and neurodegenerative disorders

Copper ions are found in all living organisms. It is an essential nutrient in humans, animals, and plants [168–171], where it plays crucial roles in redox chemistry and the actions of enzymes and proteins, especially those related to energy metabolism [169, 179]. Moreover, it is fundamental for normal brain and nervous system development, as it is involved in the synthesis of neurotransmitters and in the production and maintenance of myelin [147, 174, 175].

Copper-containing compounds have also been used in medicine for centuries. Several studies proposed that copper chelators may play a potential therapeutic role in certain inherited diseases of copper homeostasis as well as in neurodegenerative diseases, such as Parkinson’s, Wilson’s, Menkes, Alzheimer’s [147, 177], and prion diseases [172, 173, 180, 181]. Various treatments for prion disease have been trialed. One was based on the use of the copper chelator _D_-penicillamine (_D_-PEN), which demonstrated a delay in the onset of prion disease in mice [182, 183]. In another trial, a significant delay in the onset of prion disease was observed when scrapie-infected hamsters were treated with copper. Copper ions inhibit in vitro conversion of prion protein into amyloid fibrils [20]. In cross-correlation analyses, it showed an antiaggregatory effect [184–186]. Altered levels of copper and manganese ions have been observed in prion-infected brain tissues [182].

Copper can have one of several oxidation states, including Cu (IV), Cu (III), Cu (II), and Cu(I); the most common states are Cu(I) and Cu (II) [187–189]. The coordination chemistry of Cu(I) is different from that of Cu (II), because Cu(I) complexes usually have a lower coordination number (CN) than C (II) complexes have. For example, Cu(I) complexes are usually tetrahedral or square planar with CN = 4, trigonal with CN = 5, or linear with CN = 2, whereas Cu (II) complexes usually have higher coordination numbers (primarily octahedral, with CN = 6). The large number of possible combinations of copper ions allows for a wide range of copper coordination complexes, ranging from monodentate to hexadentate [190–192]. Importantly, Cu(I) has d^10^ configurations and forms colorless and diamagnetic compounds, whereas Cu (II) has d^9^ configurations and forms colored and paramagnetic compounds [10, 11, 193–203]. There is an important difference in stability of Cu(I) and Cu (II) ions that strictly defines their biological role in the living organisms. The low stability of Cu(I) has led to the relative scarcity of studies on the biological roles of Cu(I) [39], whereas the higher stability of Cu (II) has led to extensive examination of its biological role in neurodegenerative disorders [204].

Studies have highlighted the role of Cu (II) ions in synaptic transmission, axonal targeting, neurite outgrowth as well as in the modulation of signaling cascades induced by neurotrophic factors. Copper not only modulates neurotransmitter receptors at synapses but it can also affect the trafficking of synaptic vesicles and modulate the interaction between proteins involved in secretory vesicle pathways [205]. Copper is clearly important in the normal development of the brain and nervous system [206–211]. It follows that copper deficiency can lead to nervous system degeneration. A decrease in copper from its normal levels can lead to several neurodegenerative and other diseases where aggregation of proteins plays a crucial role [187, 212]. On the other hand, an excessive amount of copper (applicable especially to Cu (II) ions) can lead to cytotoxicity, owing to the ability of Cu (II) to initiate redox cycling and to produce reactive oxygen species (ROS) [213, 214].

The biological function of copper ions in many copper-binding proteins and enzymes involves changing copper’s oxidation state through various redox reactions (e.g., Fenton and Haber Weiss reactions).

1 $$ {\mathrm{Cu}}^{2+}+{\mathrm{O}}^{2-}\to {\mathrm{Cu}}^{+}+{\mathrm{H}}_2{\mathrm{O}}_2 $$

2 $$ {\displaystyle \begin{array}{l}{\mathrm{Cu}}^{+}+{{\mathrm{O}}_2}^{-}{\mathrm{Cu}}^{2+}\left({{\mathrm{O}}^{2-}}_2\right)\to {}^{2\mathrm{H}+}{\mathrm{Cu}}^{2+}+{\mathrm{H}}_2{\mathrm{O}}_2\\ {}\mathrm{C}{\mathrm{u}}^{2+}+{\mathrm{O}}^{2-}\to {\mathrm{Cu}}^{+}\ {\mathrm{H}}_2{\mathrm{O}}_2\end{array}} $$

The enzyme superoxide dismutase 1 (SOD1) is present in almost all eukaryotic cells and a few prokaryotic cells that contain both copper and zinc [40–42, 215]. SOD1 catalyzes the dismutation of the superoxide radical to hydrogen peroxide and oxygen [43–49]. This catalytic cycle is, however, beyond the scope of this review.

Interestingly, amyloid precursor protein (APP), which is found in Alzheimer’s disease patients, can reduce Cu (II) to Cu(I) in a cell-free system [11]; moreover, Cu (II) can be reduced to Cu(I) and remains bound to APP [10, 22, 41, 42, 50, 51]. This suggests that PrP is a target of copper-catalyzed oxidation and that this reaction leads to profound structural changes in the protein. Oxidation therefore must be taken into account as a potential side reaction when considering the role of copper in prion disease [52, 53, 202, 203].

In summary, copper is known to play an important role in neurological development. It can lead to neurodegenerative disorders when present in excess or deficient levels. This suggests that prion diseases may also be affected by the availability or lack of availability of copper in the brain.

## The biological roles of copper ions in neurodegenerative disorders

Mis-folded protein aggregates have been associated in several neurodegenerative disorders including Parkinson’s, Alzheimer’s, and prion disease [54]. Proteins’ aggregation rate depends on protein concentration and the ratio of the presence of metal ions like Cu^2+^, Zn^2+^, Co^2+^, Cr^3+^, and Ni^2+^ [55–57]. Tau protein (TP) and α-Synuclein are examples of biomolecules whose aggregation rates are dependent on their concentration and the metal ion coordination properties [58–60]. TP aggregation found in the neuronal cells of Alzheimer’s disease patients [59, 60] while α-Synuclein aggregation is associated with Parkinson’s disease progression [58].

TP found in the neuronal cells of the central nervous system and its aggregation is associated with Alzheimer’s disease [61]. TP is a neuronal microtubule-associated protein and plays a key role in microtubule stabilization in neuronal cells [62]. In general, TP aggregation initiated when the protein gets into the hyperphosphorylated form, which could result in microtubule (MT) assembly decomposition [54, 63]. TP aggregation is promoted in the presence of high Cu^2+^ concentration in the brain [61, 64]. A number of binding sites of Cu^2+^ with TP have been reported [65–67]. This binding leads to activation of GSK3β kinase [68] or activation of CDK5 [64] supporting the progress of Alzheimer’s disease [69].

α-Synuclein protein abundant in the brain, mainly in the presynaptic terminals and is involved the release of neurotransmitters, regulates glucose and dopamine level [70, 71]. Misfolded α-synuclein aggregation is the major component of Lewy neurites (LNs) and Lewy bodies (LBs), which are pathological hallmarks of Parkinson’s disease and other neurodegenerative synucleinopathies [72–74]. The aggregation rates of α-synuclein affected by many factors for instance α-synuclein concentration, pH, post-translational modifications (PTM), and metal ions as Cu^2+^, Zn^2+^, Al^3+^, Fe^3+^, Ca^2+^, and Mg^2+^ [75, 76]. High Cu^2+^ concentrations have been reported in the cerebrospinal fluid of Parkinson’s disease patients [77], which accelerates the aggregation rate by promoting the nucleation [69, 78]. α-Synuclein–Cu (II) complexes formed through a high-affinity copper-binding site or low-affinity copper-binding sites. The high-affinity of copper-binding sites is located at the *N-*terminus with residues Met1, Asp2, and Met5. The low-affinity copper-binding sites are located at the *N-*terminus residue His50 or at the C-terminal part with residues Asp119, Asp121, Asn122, and Glu123 [78–80].

In summary, association between Cu^2+^ and its effect on protein aggregation had been repeatedly reported [64, 67, 78]. Misfolding protein aggregations are common in many neurodegenerative diseases. This suggests that Parkinson and Alzheimer’s diseases may also be affected by the availability or lack of availability of copper in the brain.

## Roles of copper ions in prion diseases

PrP is known to bind copper ions, and this binding interaction may affect PrP^C^ function and its conformational transformation to the PrP^Sc^ form. However, there is contradictory evidence concerning whether copper ions are beneficial or deleterious to the development of prion diseases [81–85].

Both in vitro and in vivo evidence has been reported for PrP binding to copper ions. Hornshaw et al. showed the first link between copper and prion proteins in 1995 [86] in an investigation of the binding between different transition metals and synthetic peptides. They hypothesized that copper ions bind to the N-terminal octapeptide tandem repeat sequence that corresponds to three or four copies of human PrP (PHGGGWGQ) [87–89]. Although Hornshaw et al. conducted in vitro experiments, their results suggested that PrP might be a copper-binding protein in vivo and that PrP preferentially binds copper over other metals [90, 91, 180]. Another study showed that copper ions bind to His96 and His111 of wild-type PrP at pH 5.5, whereas it interacts with His111 at pH 7.5 [202]. Pathological point mutations alter copper coordination under acidic conditions and metal is then anchored to His111 [202]. Additional studies have since confirmed that PrP specifically and preferentially binds copper compared with other transition metals [92, 93]. Some reports have claimed that interaction with Mn causes conversion of PrP^c^ to PrP^res^, as detected by in vitro studies. Near-infrared spectroscopy coupled with multivariate analysis suggested that (i) PrP binds both Mn and Cu differently, (ii) PrP-Cu, and not PrP-Mn, protects the metal from the water, increasing protein stability, and (iii) PrP-Cu remains stable in solution, whereas PrP-Mn undergoes changes leading to fibril formation [94].

Later studies have shown that the binding of copper to PrP can affect its conformational transition to the infectious form. Takeuchi et al. (1996) showed that PrP requires copper to remain “normal” and non-infective. They suggested that a lack of copper might contribute to prion diseases [95, 96]. A similar study showed that the interaction of Cu (II) ions with PrP promotes a shift from a predominant α-helical structure of PrP^**C**^ to the β-sheet structure of the infectious isoform, PrP^Sc^, thus suggesting a negative role for copper ions in disease onset [97]. The results do not support Takeuchi’s proposal that the interaction of copper with prion proteins may lead to conformational changes (formation of an α-helical structure on the C-terminal side) that prevent aggregation. Zheng et al. studied the impact of the G127V mutation on the structural and dynamical properties of PrP using NMR and molecular dynamic methods [189]. They concluded that replacement of G127 by V destabilizes the β-sheet and affects the geometric stacking of the α-helices inside the prion molecule.

Studies performed in cell culture models and animal models have provided evidence both for and against the role of copper in promoting the development of prion diseases. For example, several studies have shown that copper functions as an antioxidant agent in copper-containing PrP, which enhances neuronal survival [98]. In contrast, Hijazi et al. found that copper plays a protective role in prion diseases, as they observed a significant delay in prion disease onset in scrapie-infected hamsters treated with copper ions, whereas administration of copper ions to normal hamsters promoted cerebellar PrP^C^ accumulation [12, 39, 99–112, 202]. Moreover, the accumulation of the disease-related conformation (PrP^Sc^) is significantly decreased in scrapie-affected neuroblastoma cells cultured in the presence of copper. On the other hand, normal neuroblastoma cells cultured in the presence of copper exhibited inhibition of the internalization of PrP^Sc^ [113]. In agreement with this result, Toni et al. reported that copper modifies PrP^C^ expression and pathways in cultured neurons and that PrP mRNA expression in GN11 neurons is significantly decreased by the addition of copper ions at physiological concentrations [114]. These results suggest that extracellular copper can be used to control the amount of cellular PrP and may be an effective strategy to decrease the expression of PrP^C^, consequently decreasing the possibility of its conversion to the pathological isoform PrP^Sc^ [115].

The contradictory results from the studies described above indicate that the role played by copper in the development of prion diseases is unclear. Further research is needed to resolve these contradictions. Structural biology approaches, in general, and NMR spectroscopy, in particular, have the potential to be very useful in the study of copper ion coordination with PrP to help elucidate the role played by copper ions in prion diseases [116].

## NMR spectroscopy

NMR spectroscopy is a powerful analytical tool. It is able to differentiate the unique magnetic environment of a nucleus in a single molecule’s various positions at the atomic level [117, 118]. Moreover, NMR can be used in structural elucidation as well as for kinetics and thermodynamics studies [99, 119, 120]. Most importantly, NMR provides information on the environment of specific atom sites and their neighboring attached atoms using in two dimensions [108, 121]. Thus, NMR spectroscopy is extensively used in a wide range of applications, including organic chemistry [108], biochemistry, polymer chemistry [122], inorganic chemistry [122], structural biology [52], physics [61, 123–127], biology, and drug discovery [52, 128, 129]. Through NMR experiments, researchers can study samples in the solid state [130–132], gel phase [133–136], tissue state [137–139], gas phase, and solution state [140–143]; these approaches have been used to investigate molecular structures, concentration levels, and molecular dynamics [144–146]. Moreover, the continuous development of NMR experimental methods and NMR machinery, such as dynamic nuclear polarization (DNP) and high-field NMR spectrometers, has continuously enhanced research on the physical and chemical properties of samples [216–218].

The main disadvantage of NMR spectroscopy is its low sensitivity, making milligrams of a sample necessary for useful NMR measurements. The low natural abundance of both ^15^N and ^13^C also has to be overcome in the application of NMR spectroscopy to biological samples, such as in the study of proteins. Proteins isotopically labeled with ^13^C and/or ^15^N are therefore often used in protein NMR experiments. NMR spectroscopy uses many multidimensional approaches to resolve protein structures, their dynamics and to enhance the resolution of complicated NMR spectrum [219–223].

There are also several limitations to the use of NMR spectroscopy as an analytical tool to study the interaction between copper ions and prion proteins. Generally, paramagnetic ions such as Cu (II) cause a significant broadening in the NMR resonance even at a very low concentration, and this broadening hinders NMR studies at a stoichiometric ratio. Consequently, NMR studies of PrP are typically performed at low copper-ion-to-PrP ratios. Diamagnetic Cu(I) ions that facilitate the use of NMR studies at higher copper-to-PrP ratios are unstable compared with Cu (II) ions and can be easily oxidized to Cu (II) under physiological conditions. However, this problem can be overcome by adding reducing reagents to the NMR tube under inert conditions and then sealing the NMR tube to prevent oxidation.

### Two-dimensional NMR spectroscopy

NMR experiments are not only limited to the one-dimensional (1D) space. They can be extended to different types of multidimensional approaches. Two-dimensional (2D) NMR spectroscopy can be used for many applications including molecule identification and structural elucidation, as has been done for PrP and their biologically important complexes with transition metals and other proteins [224]. In general, 2D NMR can be used to overcome the problem of overlapping resonances by dispersing the overlapping chemical shift in a second dimension. The additional resolution offers a practical solution to detecting and identifying specific sites within macromolecule, as in the case of Cu (II) ions [223]. Such identification is not possible with the 1D approach. For example, various homo-nuclear 2D ^1^H-^1^H-NMR experiments, including total correlation spectroscopy (TOCSY) [225–234], correlation spectroscopy (COSY) [219, 234–241], and heteronuclear experiments such as ^1^H,^13^C-single quantum coherence (^1^H-^13^C-HSQC) and heteronuclear multiple bond correlation (HMBC) have been routinely used in to assign protein signals and to study protein interactions with ligands in drugs and small molecules [242]. Here, we present heteronuclear single-quantum coherence spectroscopy (HSQC) as an example of the most powerful approaches used to assign signals and to probe ligand protein interactions [243]. HSQC is a type of through-bond correlation spectroscopy that utilizes heteronuclear correlations and enhancement of the signal coming from the nucleus of lower sensitivity, such as ^13^C or ^15^N by transferring the nuclear spin polarization from the more sensitive nucleus (usually ^1^H) via J-coupling. The general output of HSQC is 2D spectra of the chemical shift of one nucleus, such as ^1^H, which is usually detected in the directly measured dimension, and the chemical shift of the other nucleus, such as ^13^C, which is recorded in the indirect dimension. The ^1^H,^13^C-HSQC spectrum coordinates the chemical shift of protons and nitrogen or carbon atoms that are directly covalently bonded, providing only one cross peak for each H-N or H-C coupled pair. Thus, HSQC is useful for the assignment of the protein backbone and side-chain NH signals are assigned by ^1^H,^15^N-HSQC. Moreover, utilizing the sensitivity of the ^1^H atom is an effective approach to reducing the experimental time for nuclei with low natural abundances and/or sensitivities, such as ^15^N and ^13^C. The experimental time necessary for HSQC experiments is usually shorter than for ^1^D, ^13^C, and ^15^N NMR experiments. Indeed, HSQC was used to study the interaction of copper with PrP [52, 123, 219, 244–246].

## NMR studies of Cu(I) and Cu (II) ions-prion interactions

**Fig. 4:**
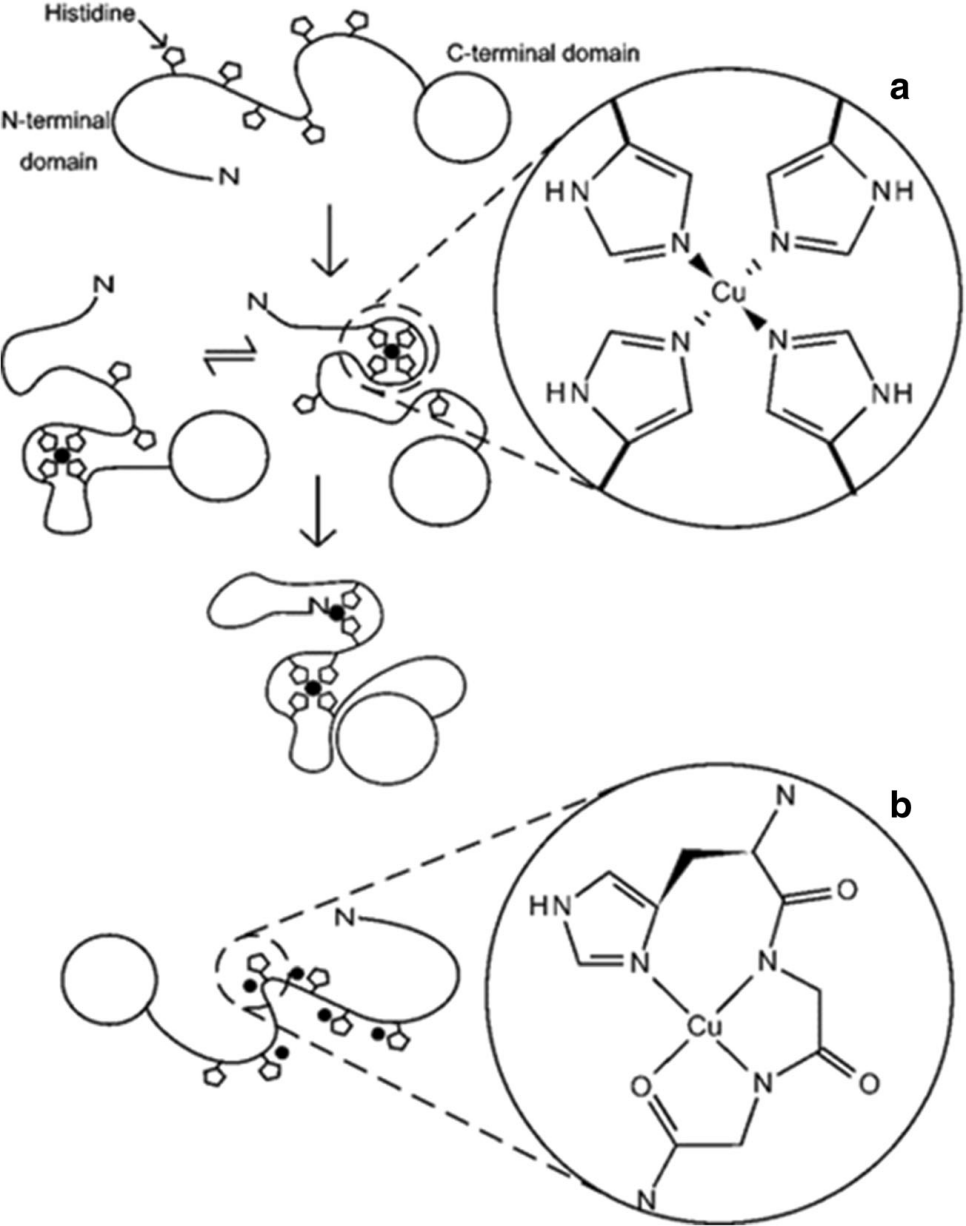
Various binding models of Cu (II) ion coordination with the full-length prion protein at **a** pH 5.5 and **b** pH 7.4, demonstrating the coordination by an exchange of histidine imidazoles. From [249] with permission from Portland Press Ltd

NMR is the method of choice for studying protein structures and dynamics and for investigating protein-metal ion interactions [247]. The protein binding sites for paramagnetic species such as Cu (II) ions can be examined by monitoring the line broadening of NMR resonance signals; the signals of the protein binding sites are more affected than are other signals. Indeed, NMR spectroscopy was used frequently to study the interaction of copper with PrP [248]. For example, Wells et al. used NMR to investigate how Cu (II) ions interact with the full length of PrP under acidic conditions at pH 5.5. The results showed that the protein binds with two copper ions while all six histidine residues in the unfolded N-terminal act as ligands (Fig. 4) [41, 246, 247, 250–252]. The interaction between a diamagnetic ion such as Cu(I) and other molecules such as proteins can be observed by monitoring the ordinary chemical shift change (change of the location of the cross-peak on the spectrum) and the interaction causing a change in the chemical shift value of nuclei within residues of the binding site. Indeed, detecting the interaction between Cu(I) and proteins has become a common approach [41, 247, 252], and the interaction between Cu(I) ions and PrP has been successfully studied using NMR spectroscopy techniques [253–255]. Taking into account the fact that Cu(I) is diamagnetic, NMR studies of its complexes with prions could be easier and more accurate because Cu(I) ions do not cause signal broadening [116].

**Fig. 5:**
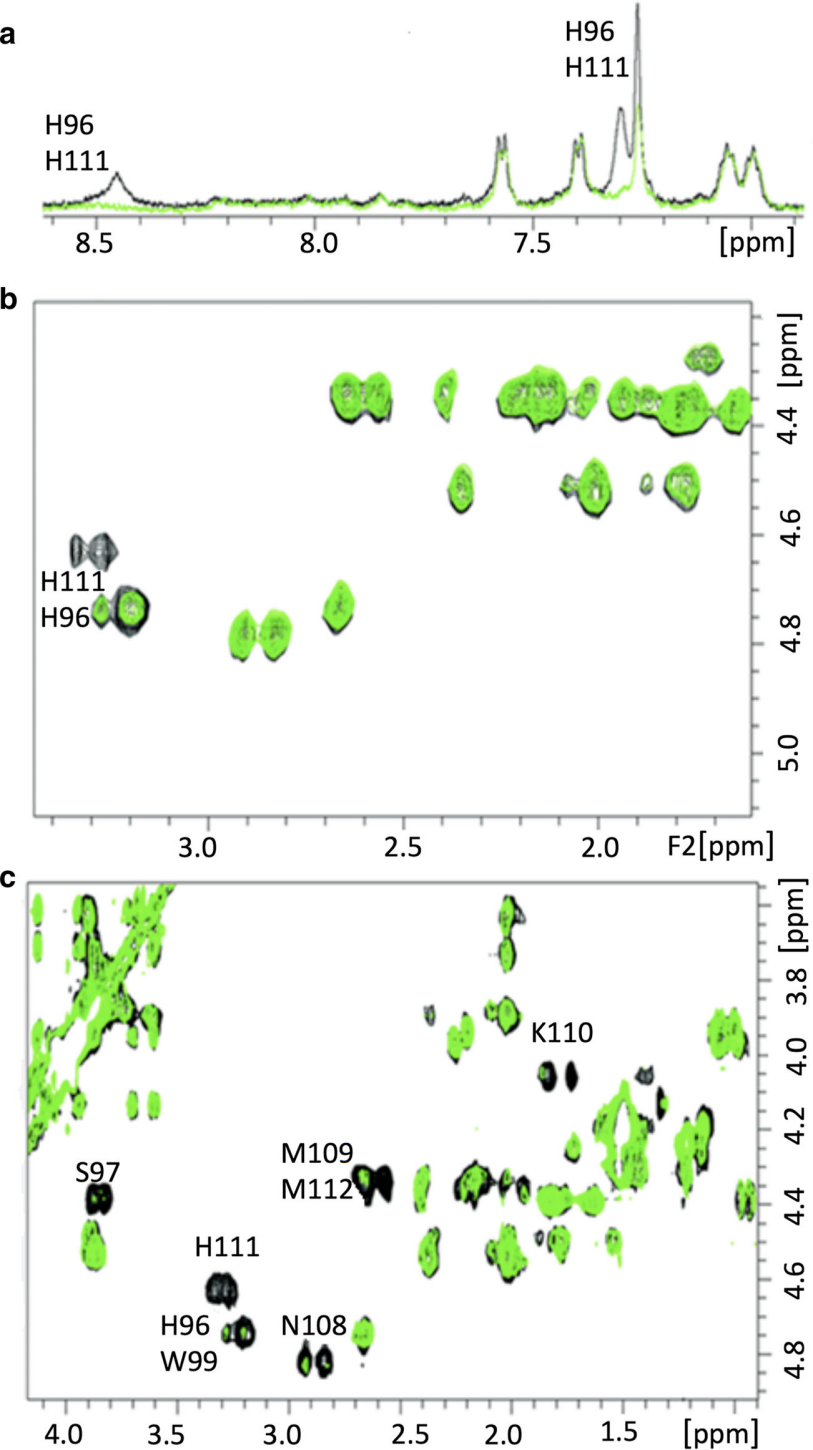
**a** Aromatic region of the 1H 1D NMR spectra of hPrP91–127 at pH 7.2 in the presence of 40 mM sodium dodecyl sulfate (SDS) in the absence (black) and presence (green) of Cu2+. **b** 2D TOCSY spectra of the aliphatic region with a copper-to-ligand ratio of 1:10. **c** The aliphatic region of the 1H–1H 2D TOCSY spectra with a metal-to-ligand ratio of 1:1.1 [231, 260–265]

Various NMR spectroscopy approaches have been utilized to study the interaction of copper with PrP. Recently, ^1^H-^15^N HSQC NMR spectroscopy was employed to study the interaction between the PrP’s copper-bound octarepeat domain [249, 256–259]. The results suggest a molecular foundation for the role of copper in mediating the cis interaction in prion proteins and suggest that the global domain can regulate the N-terminus, whereas the disruption of the cis-interaction occurs by mutation or by direct competition with globular domain ligands, contributing to protein dysregulation and prion disease [52, 260–267]. ^1^H NMR has been used to study the interactions of copper with different peptides corresponding to PrP, including 2-, 3-, and 4-octarepeat sequences [265]. The resulting NMR spectra show a clear broadening of the histidine ^1^H residues in each octarepeat coordinated with the Cu (II) ion, with the four octarepeat peptides cooperatively binding to four Cu (II) ions. Two-dimensional ^1^H-^1^H TOCSY NMR spectroscopy has been used to study the interaction between copper and the residue 91–127 fragment of the human prion protein (hPrP) [268–273]. In agreement with previous results, NMR spectra from that study show that copper ions selectively bind His-96 and His-111 (Fig. 5) [274, 275]. Interestingly, the results confirm that the protein undergoes a conformational change after binding Cu (II) ions in the presence of sodium dodecyl sulfate (SDS) micelles; the binding strongly stabilizes the α-helical conformation of the peptide backbone [202]. Some researchers hypothesize that copper binding to the prion protein can be protective against the conversion of the protein to its infectious form [260].

NMR spectroscopy has also been used to analyze the interactions between copper and PrP at different pH values. The interaction of Cu (II) ions with full-length PrP has been investigated under mildly acidic conditions. The results show that two Cu (II) ions bind all six histidine residues of the unfolded N-terminal domain and the N-terminal amine coordinate as ligands [260]. Different copper-protein coordination models have been reported under different pH conditions [202, 276–279]. For instance, at pH 7.4, PrP may interact with a fifth or even sixth coordination site in the flexible region between the octarepeats and the PrP globular C-terminal domain involving His96 and His111 [280]. Similar studies have shown that PrP binds between five and six equivalent units of Cu (II) at pH 7.4, indicating that the interaction of copper with PrP is highly dependent on pH [280]. These reports have proposed that PrP functions may be associated with its ability to bind copper in a pH-dependent fashion [280].

Although the coordination geometry of the different copper-PrP interaction models [281] has been the focus of many studies, surprisingly few investigations have focused on the structural changes induced by the binding of Cu(I) to PrP [265]. Badrick et al. investigated the interaction between Cu(I) ions and the hPrP by using both 1D and 2D ^1^H NMR experiments. The results show that Cu(I) interacts with PrP in a manner different from that of Cu (II), with the Cu(I) interaction representing a tetrahedral model in which copper coordinates with two imidazoles attached to His96 and His111 and two sulfurs (Met109 and Met112) [282, 283]. The interaction between the copper ions and Met residues has sparked a strong debate in the literature concerning possible direct binding with sulfur atoms [284]. Several reports have ruled out the possibility of copper interacting with Met109 or Met112 [285–287]. However, Shearer et al. demonstrated that copper interacts with both Met residues in PrP under mildly basic conditions [265, 284]. These contradictory results may be explained by considering the factors that might lead to different copper-PrP coordination models. Different factors should be considered in explaining that different copper-prion interactions, such as the pH, copper oxidation state, and copper/protein ratio, may lead to different copper-PrP coordination models. For example, the copper oxidation state is a very important factor that determines copper complexation because Cu(I) normally adopts a tetrahedral coordination geometry, whereas Cu (II) prefers an octahedral or square planar coordination geometry [288, 289]. Cu(I) ions can be oxidized simply to Cu (II), and Cu (II) can be reduced to Cu(I), thus enabling copper to be involved in electron transfer reactions and copper-protein interactions and potentially leading to conformational changes associated with changes in the oxidation state [290]. To elucidate the role of copper in prion diseases, further investigations should be conducted to study the relationship between electron transfer reactions and the conformational transformation associated with copper-protein interactions.

## Conclusion

Prion diseases are a group of fatal neurodegenerative disorders that occur when prion proteins change their conformation from the normal PrP^C^ form to the disease-specific PrP^Sc^ structure. These diseases affect both humans and animals. Animals acquire prion diseases from contaminated feed or other animals, whereas humans can contract prion diseases genetically, sporadically, or via acquisition from infected animals and humans. Although the disease pathology is not completely understood, there is general agreement that the abnormal disease-associated protein conformation (PrP^Sc^) causes prion diseases through the degeneration of the nervous system and leads to death at an advanced stage. It has been repeatedly reported that copper ion may play a major role in structural conversion from a healthy (native) α-helix rich PrP isoform to the predominantly β-sheet conformation (PrP^Sc^). The conversion could be developed by the exposure of the protein to high concentrations of Cu (II) ions.

It is well established that an excessive amount of copper (especially Cu (II) ions) can lead to cytotoxicity, owing to the ability of Cu (II) to initiate redox cycling and produce reactive oxygen species (ROS). However, despite the wide range of studies on copper interaction with prion proteins, the mechanisms by which Cu (II) ions induced protein misfolding and aggregation remains unknown.

The proper application of the NMR spectroscopy techniques could lead to better insight if the studies include both protein function and structure. A gradual titration of prion proteins with different concentration levels of Cu (II) ions could lead to the most optimal concentration as we believe like other bioactive molecules with low or high concentrations could lead to abnormal conditions. To evaluate the copper redox effects, it is also important to study the interaction of PrP with different copper oxidation states, particularly ion (I\II) interactions. The NMR spectroscopy offers atomic-level insights into the interactions of copper ions (I\II) with PrP under physiological conditions (like pH ~ 7.4), enabling researchers to study the role played by copper and other ions in the progress of the prion disease.

## Electronic supplementary material

ESM 1 (DOCX 22 kb)

## Abbreviations

1D: One-dimensional
1H-13C-HSQC: 1H,13C-single quantum coherence
2D: Two-dimensional
APP: Amyloid precursor protein
CJD: Creutzfeldt-Jakob disease
COSY: Correlation spectroscopy
Cu(2+): Copper (II) ions
DNP: Dynamic nuclear polarization
D-PEN: D-penicillamine
FFI: Fatal familial insomnia
GPI: Glycosylphosphatidylinositol
GSS: Gerstmann-Sträussler-Scheinker syndrome
HMBC: Heteronuclear multiple bond correlation
HSQC: Heteronuclear single-quantum coherence spectroscopy
NMDAR: N-methyl-d-aspartate receptors
NMR: Nuclear magnetic resonance
PrP: Prion protein
PrP^C^: Normal prion protein
PrP^Sc^: Disease-associated isoform pf prion protein
ROS: Reactive oxygen species
SDS: Sodium dodecyl sulfate
SOD: Superoxide dismutase
SOD1: Enzyme superoxide dismutase 1
TOSCY: Total correlation spectroscopy
