# Supplementary material for: Using NMR spectroscopy to investigate the role played by copper in prion diseases
Source: Neurol Sci. 2020 Apr 24;41(9):2389–406. doi: 10.1007/s10072-020-04321-9 (PMC7419355; doi:10.1007/s10072-020-04321-9)
Supplement: Supplementary file 1 — (DOCX 22 kb) [file 10072_2020_4321_MOESM1_ESM.docx]

**Table S1** Human prion diseases

|  | **Creutzfeldt-Jakob Disease, classic (CJD)** | **Variant CJD** | **Gerstmann-Straussler-Scheinker Syndrome (GSS)** | **Fatal Familial Insomnia (FFI)** | **Kuru** |
| --- | --- | --- | --- | --- | --- |
| **Cause** | Exposure to material contaminated with CJD prions | Consumption of material from cattle infected with BSE | Familial (germ line *PRNP* mutation) | Familial (germ line *PRNP* mutation) | Endocannibalism |
| **Year of first incidence** | 1920 | 1994-1995 | 1936 | 1982 | 1900s |
| **Country of first occurrence** | Germany | UK | Austria | Italy | Papua New Guinea |
| **Incidence** | 1 million per year worldwide | Peaked in 1999-2000 (229 patients worldwide). No new cases after 2012 | 1 and 10 per 100 million | <1/1000000 (worldwide) | Peaked in 1950s (>200 cases/year). No new cases since endocannibalism was halted |
| **Onset** | 30 to 55 years | 27 years (range 12 to 74) | 50s (range 20s to 70s) | Late 40s | Children and young women |
| **Duration of illness** | 4 months- 2 years | 13-14 months | 60 months (range 2 to 10 years) | 18 months | 4-24 months |
| **Concentrates of autopsied brain tissues from these patients can transmit the infection to:** | Apes  Transgenic mice  Chimpanzees | Apes  Transgenic mice  Monkeys | Apes  Transgenic mice  Monkeys | Transgenic mice | Chimpanzees  Squirrel monkeys |
| **Occurrence mechanisms** | | | | | |
| **1- Genetic** | Yes | No | Yes | Yes | No |
| **2- Sporadic** | Yes | No | No | Yes | No |
| **3- Acquired** | Yes | Yes | No | No | Yes |
| **Diagnosis** | | | | | |
| **1-Clinical signs and symptoms** | Rapidly progressive dementia  Ataxia  Supranuclear gaze Paralysis  Myoclonus  Other motor features-early neurologic signs | Painful dysesthesias  Delayed neurologic signs,  Cognitive and cerebellar dysfunction  Involuntary movements | Late-onset, rapid dementia  Slowly progressive ataxia  Motor disorder  Amyotrophy | Severe progressive insomnia  Tachycardia  Hyperhidrosis  Hyperpyrexia  Motor  Late cognitive manifestations | Headache and arthralgia  Cerebellar ataxia  Voluntary tremor  Involuntary movement  Euphoria  Dementia  Loss of grasp reflexes |
| **2- Neuropathological features** | Spongiform degeneration  Astrogliosis spread through the cortex  Deep nuclei of the brain | Severe spongiform degeneration in striatum  Florid plaque  Clear neuronal loss  Gliosis within the thalamus | Multiple amyloid plaques within the cerebral and cerebellar cortices | Lack of spongiform degeneration  Presence of neuronal dropout  Gliosis within the thalamus | Absence of inflammation  Starburst amyloid plaques in the cerebellum |
| **3- Periodic sharp wave complexes on electroencephalogram (EEG)** | Often present  Tend to appear late | Often absent  Rarely reported at late stages of disease | Nonspecific slowing (not helpful in confirming the illness | Absent  Generalized slowing | Alpha rhythm slowing  Increase in theta activity  Occasional delta slowing |
| **4- MRI** | Prominent, symmetric *striatum* T2-weighted hyperintensities  Less prominent cortical ribboning  No pulvinar sign | Pulvinar sign present | Not helpful in confirming the illness | Reduced thalamic diffusion  Thalamic hypometabolism on fluorodeoxyglucose positron emission tomography (FDG-PET) imaging | Have not been described |
| **5- 14-3-3, NSE, and t-tau biomarkers** | Elevated | Less sensitive than classic CJD | Not elevated (not helpful in confirming the illness) | Very low sensitivity (not helpful in confirming the illness) | Have not been described |
| **Mutations** | | | | | |
|  | Caused by more than 20 different *PRNP* mutations including:  Missense mutation (E200K: 598A→G; 200Glu→Lys) in the prion protein (PRNP) gene  Isoleucine instead of valine (V) at position 210 to (V210I)  Asparagine instead of aspartic acid (D) at position 178 in the protein (D178N) with a polymorphism of valine in both *PRNP* genes at position 129. | Homozygous for methionine at *PRNP* codon 129  MV at codon 129, thus suggesting that codon 129 heterozygosity is also a susceptibility factor for the disease. | Caused by a several *PRNP* mutations, including:  Leucine instead of proline at position 102 (P102L)  Valine instead of alanine at position 117 (A117V)  Other mutations: P105L, P105T, Q145X, F198S, Q217R, and several OPRI mutations  Homozygosity for a polymorphism at position 129 (both residues being methionine) | Single *PRNP* point mutation  Asparagine instead of aspartic acid at position 178 (D178N) with codon 129 polymorphism [cis methionine at codon 129] | *PRNP* polymorphism at codon 127  *PRNP* Polymorphism at codon 129 where either methionine (M) or valine (V) may be encoded  *PRNP* variant—G127V polymorphism |
